# Outcome of abdominal massage before gavage feeding on tolerated feeding for low birth weight infants

**DOI:** 10.1002/nop2.1144

**Published:** 2021-11-30

**Authors:** Abdelaziz Hendy, Nahed Saied El‐Nagger, Ahmed Abozeid, Fadia Ahmed Reshia, Shahenda A. Salih, Manar Fayez Alruwaili, Ahmed Hendy

**Affiliations:** ^1^ Pediatric Nursing Department, Faculty of Nursing Ain Shams University Cairo Egypt; ^2^ College of Applied Medical Sciences Jouf University Sakākā Saudi Arabia; ^3^ Critical Care and Emergency Nursing, Faculty of Nursing Mansoura University Mansoura Egypt; ^4^ Nursing Department Faculty of Medical Technical Science ALzaiem Alazhari University Khartoum Sudan; ^5^ Nursing Department College of Applied Medical Sciences Jouf University Sakākā Saudi Arabia; ^6^ Department of Computational Mathematics and Computer Science Institute of Natural Sciences and Mathematics Ural Federal University Yekaterinburg 620002 Russia; ^7^ Medical surgical nursing department, Faculty of nursing Ain Shams University Cairo Egypt

**Keywords:** abdominal massage, low birth weight, tolerated feeding

## Abstract

**Aim:**

To assess the effect of abdominal massage pre‐gavage feeding on tolerated feeding for low birth weight (LBW) infants.

**Methods:**

An experimental research design at a government hospital at Egypt. Purposive sample composed of LBW infants was randomly divided into study and control groups each with 60 LBW infants.

**Results:**

A total of 55% of the participants in the study group grew sleepy, whereas only 15% of the studied participants in the control group grew sleepy. The abdominal circumference after feeding in the study group was 23.18 ± 2.99 cm, whereas that in the control group was 24.79 ± 2.99 cm. The gastric residual volume in the study group was 0.8 ± 0.10 ml, whereas that in the control group was 3.86 ± 1.03 ml.

**Conclusion:**

Finally, abdominal massage had a positive impact on the postfeeding state of alertness and feeding tolerance.

**What is already known about the topic?**

‐ Birth weight is an important element of future growth pattern and nutritional status in LBW.

‐ Abdominal massage is a therapeutic nursing care method that can arouse parasympathetic activity and has a positive effect on the digestive system

**What this paper adds?**

‐Pre‐gavage abdominal massage improve LBW infants' sleep

‐Abdominal massage before gavage feeding decreases the abdominal circumference after feeding in the study group than in the control group.

‐Also, decreasing gastric residual volume in the study group than that in the control group.

## 1 INTRODUCTION

Low birth weight infant (LBW infant) is defined as neonatal body weight at birth <2,500 g according to the World Health Organization (WHO) definition. LBW infant continues to be a vital general health issue worldwide and is linked with a variety of both long‐ and short‐term problems (Blencowe et al., 2019). Globally, approximately 14.6% of all births are LBW infants, resulting in approximately 20.5 million births a year. In Africa, it is estimated that 13.7% of all births are LBW, representing 5.7 million births. The goal is to achieve a 30% successful decrease in the number of LBW infants by 2025 (WHO, 2012).

LBW infants can result as a consequence of an infant being born small for gestational age and because of preterm birth. Birth weight is an important element of future growth pattern and nutritional status in LBW infants. Feeding of LBW infants includes integrative harmonization of sucking, swallowing and breathing (Brown et al., 2019).

Nutritional requirement management of these LBW infants is difficult. The mixture of high nutrient needs and gastrointestinal immaturity prompts these LBW infants to not tolerate feeds, which may cause undernutrition and later adverse outcomes, such as diminished growth of the brain, delayed cognitive ability and necrotizing enterocolitis (NEC) (Seiiedi‐Biarag & Mirghafourvand, 2020).

Feeding tolerance is verified through the ability of LBW infants safely to ingest and to digest the recommended enteral feeding without difficulties or complications or accompanied by gastrointestinal dysfunction, aspiration and infection (Soltani et al., 2020). Feeding intolerance is usually based on the assessment of gastric residual volume (GRV), color and aspect and accompanying clinical manifestations such as vomiting, abdominal distension, blood in stools, apnea with bradycardia and not gaining weight over a long time range (Saeidi Hassani, 2019).

Abdominal massage is a therapeutic nursing care method that can arouse parasympathetic activity and has a positive effect on the digestive system by enhancing intestinal peristalsis, diminishing distension of the abdomen, improving the bowel transit time and defecation times, limiting the frequency of vomiting and enhancing the sleep state. Thus, improved gastric motility results in weight gain (Lu et al., 2020; Tekgündüz et al., 2014).

## 2 AIMS

The study assessed the effect of abdominal massage pre‐gavage feeding on tolerated feeding for LBW infants, through the following:

‐ Assessing feeding tolerance indicators for LBW infants during gavage feeding.
‐ Implementing abdominal massage for LBW infants before gavage feeding in the study group.
‐ Evaluating the effect of abdominal massage pre‐gavage feeding on tolerated feeding for LBW infants.

### 2.1 Research hypothesis

**H_1_**: Abdominal massage pre‐gavage feeding has a positive effect on tolerated feeding in LBW infants.

## 3 METHODS

An experimental research design was used to conduct the study from February 2020 to November 2020. This study was conducted at the neonatal intensive care unit (NICU) at a government hospital at Egypt. A purposive sample of infants was composed of LBW infants who received care at the previously mentioned setting. The first 60 LBW infants who are newly admitted to the NICU were randomly assigned to the “control group” and the next 60 LBW infants who were newly admitted to the NICU after control group infants were allocated to the “study group.” The inclusion criteria were gestational age of 30–36 weeks, birth weight 1,000–2,500 g and dependent on gavage feeding during the time of intervention; infants who suffered LBW from major health problems (i.e., NEC and serious infectious diseases), a congenital anomaly or with a brain injury and needing surgery were excluded.

### 3.1 Sample size

The sample size was calculated based on the study carried out by Ghasemi et al. (2019). Based on the mean GRV (4.25 and 2.43) and *SD* (1.74 and 1.9) related to premature infants admitted to the NICU, statistical power of 85%, confidence level (1‐Alpha Error) 95%, Alpha 0.05 and Beta 0.15, the sample size determined for each group was at least 20 LBW infants.

### 3.2 Tool containing three parts

**Part I:** Characteristics of LBW infants such as sex, weight, gestational age and type of feeding.

**Part II:** State of alertness such as crying, quiet and sleeping.

**Part III:** Indicators of feeding tolerance, such as the frequency of defecation/day, frequency of vomiting and abdominal distention/day, abdominal circumference after feeding for 30 min and GRV before feeding.

### 3.3 Field work

The abdominal massage intervention for the study group is conducted by the physical therapist 30 min before gavage feeding, for 10 min three times per day for 4 days. The LBW infants must be in the supine position with the head elevated and the technique of abdominal massage is then applied (Ardiansyah et al., 2020). Abdominal massage was performed in a clockwise direction over the intestines on the wall of abdomen, push both sides of the abdomen toward the umbilical cord, from the right lower abdomen to the left lower abdomen, drawing a small circle over the entire abdomen, using the fingers and palm, making a smooth circle and sweeping down to the leg.

For the study and control groups, the researcher measured the abdominal circumference with a measuring tape after gavage feeding for 30 min and observed and palpated the abdomen of the LBW infants for distention. GRV was checked through an open Ryle tube with a drainage bag before feeding for 15 min. For any related defecation and vomiting, the researchers observed and recorded the times of and frequency per day. In addition, the LBW infant's state of alertness was checked after feeding for 10 min, and the findings were recorded.

Five neonatal nursing experts determined the content validity. Reliability of the index was evaluated with Cronbach's alpha and was 0.799.

Data collected were coded and entered through personal computer. Computerized collected data entry and statistical analysis were performed by the Statistical Package for Social Sciences version 24. Data are presented as numbers/percentages and the mean and *SD*. The T test used for comparing means among two groups and *p* < .05 was considered statistically significant.

### 3.4 Ethical considerations

Researchers conducted the research under the deliberation of the Research Ethics Review Committee. Consent was obtained from the parents of the LBW infants in the experimental study group after clarification of the study's aim, how to apply to the study and ensuring them about the confidentiality of the data collected.

## 4 RESULTS

Table 1 shows that 65% and 55% of the participants in the study group and the control group were male and female, respectively, without any significant difference between the studied groups. In addition, the mean gestational age of the study group was 32.8 ± 2.01 weeks, whereas that of the control group was 32.7 ± 1.99 weeks, and no significant difference was detected between the two studied groups (*p* > .05). The mean birth weight in the study group was 1.695 ± 0.299 kg, whereas that in the control group was 1.726 ± 0.352 kg, with no significant difference between the studied groups (*p* > .05).

**TABLE 1:** Demographic Data of Studied Groups (*N* = 60)

Characteristics	Study		Control		*T* test
	*N*	%	*N*	%	*p* value
Sex					
Male	39	65	27	45	3.998 < 0.05
Female	21	35	33	55	
Gestational age					
30–<32	21	35	21	35	1.340 > 0.05
32–<34	24	40	27	45	
34–36	15	25	12	20	
$\bar{x}$ ±	32.8 ± 2.01		32.7 ± 1.99		
Birth weight					
<1.500 Kg	18	30	15	25	1.011 > 0.05
From 1.500 < 2.000 Kg	36	60	33	55	
From 2.000–2.500 Kg	6	10	12	20	
$\bar{x}$ ±	1.695 ± 0.299		1.726 ± 0.352		
Type of feeding					
Breast milk	21	35	24	40	1.102 > 0.05
Breast milk & formula	24	40	18	30	
Formula	15	25	18	30	

**FIGURE 1:**
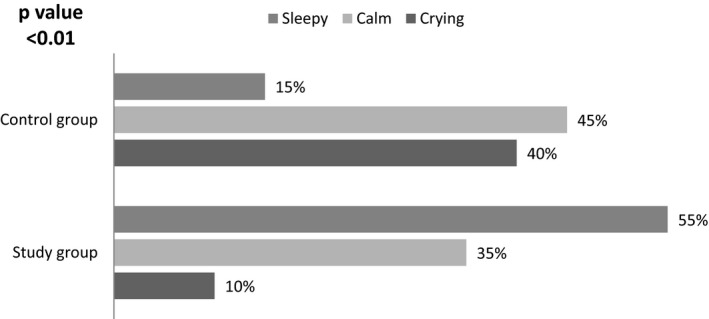
Distribution of studied groups of LBW infants according to postfeeding alertness (*N* = 60)

Figure 1 reveals that 55% of studied participants in the study group grew sleepy and 35% of them were calm after feeding. However, only 15% of the studied participants in the control group grew sleepy, and 40% of them were crying post feeding, and this difference between the groups was highly statistically significant (*p* < .01).

Table 2 shows that the mean frequency of defecations per day in the study group was 2.40 ± 0.62 times, whereas that in the control group was 1.31 ± 0.73 times with a statistically significant difference between two groups (*p* < .05). The relative frequency of vomiting per day in the study group was 0.30 ± 0.08 times, whereas that in the control group was 0.81 ± 0.24 times, and this difference was statistically significant (*p* < .05). The frequency of abdominal distention per day in the study group was 0.25 ± 0.06, whereas that in the control group was 0.72 ± 0.12 and was a statistically significant difference (*p* < .05). The abdominal circumference after feeding in the study group was 23.18 ± 2.99 cm, whereas that in the control group was 24.79 ± 2.99 cm, and this difference was statistically significant (*p* < .05). The GRV in the study group was 0.8 ± 0.10 ml, whereas that in the control group was 3.86 ± 1.03 ml, and the difference between the two groups was statistically significant (*p* < .05).

**TABLE 2:** Mean variable difference between low birth weight infants in the studied groups according to feeding tolerance on the fourth day postintervention (*N* = 60)

Feeding Tolerance Indicators	Study *N* = 20	Control *N* = 20	*T* test	*p*
Frequency of defecation/day	2.40 ± 0.62	1.31 ± 0.73	4.03	<.05
Frequency of vomiting/day	0.30 ± 0.08	0.81 ± 0.24	5.712	<.05
Frequency of abdominal distention	0.25 ± 0.06	0.72 ± 0.12	5.488	<.05
Abdominal circumference	23.18 ± 2.99	24.79 ± 2.99	3.991	<.05
Gastric residual volume	0.8 ± 0.10	3.86 ± 1.03	6.076	<.05

Table 3 indicates that the mean frequency of defecations per day in the study group pre‐intervention was 1.26 ± 0.68 times and postintervention was 2.40 ± 0.62 times and was statistically significantly different (*p* < .05). The relative frequency of vomiting per day in the study group pre‐intervention was 0.77 ± 0.19 times and postintervention was 0.30 ± 0.08 and was statistically significantly different (*p* < .05). The frequency of abdominal distention per day in the study group pre‐intervention was 0.79 ± 0.17 times and postintervention was 0.25 ± 0.06 times and was statistically significantly different (*p* < .05). Concerning abdominal circumference after feeding in the study group pre‐intervention was 25.12 ± 1.86 cm and postintervention was 23.18 ± 2.99 cm and was statistically significantly different (*p* < .05). Concerning the mean of GRV in the study group was 2.49 ± 1.14 ml and postintervention was 0.8 ± 0.10 ml and was statistically significantly different (*p* < .05).

**TABLE 3:** Mean variable difference between low birth weight infants in the study group according to feeding tolerance on the pre‐intervention and 4 days postintervention (*N* = 60)

Feeding Tolerance Indicators	Pre *N* = 20	Fourth day *N* = 20	*T* test	*p*
Frequency of defecation/day	1.26 ± 0.68	2.40 ± 0.62	4.053	<.05
Frequency of vomiting/day	0.77 ± 0.19	0.30 ± 0.08	4.869	<.05
Frequency of abdominal distention	0.79 ± 0.17	0.25 ± 0.06	5.030	<.05
Abdominal circumference	25.12 ± 1.86	23.18 ± 2.99	5.964	<.05
Gastric residual volume	2.49 ± 1.14	0.8 ± 0.10	7.169	<.05

## 5 DISCUSSION

Researchers were keen to select the samples that met the predetermined criteria, and the current results revealed that there was no statistically significant difference between the study and control groups in birth weight, gestational age, sex and type of feeding (*p* > .05). Therefore, the two study groups were similar.

After analyzing and interpreting the collected data and related states of alertness after feeding, the current study demonstrated that more than half of the LBW infants in the study group grew sleepy and more than one‐third of them were calm after feeding. However, less than one‐fifth of the studied LBW infants in the control group grew sleepy and two‐fifths of them were crying after feeding, and this difference was highly statistically significant (*p* < .01). These results are consistent with the study by Arbianingsih et al., 2020, who stated that massage is effective in limiting sleep problems in infants mainly in the domain of starting and maintaining sleep and are in accord with Wahyuni et al., 2020, who reported that there was a significant positive effect of giving infant massage on the quantity of sleep. In addition, Baniasadi et al. (2019) detected that massage had a positive effect on behavioural response and sleep pattern.

Moreover, regarding feeding tolerance, the current results demonstrated that there was a slight significant improvement in all items of feeding tolerance for the study group after abdominal massage, and there was also a slight significant difference between the study and control groups at the fourth day after abdominal massage related to all items of feeding tolerance such as frequency of defecation (2.40 ± 0.62 in the study group and 1.31 ± 0.73 in the control group), frequency of vomiting/day (0.30 ± 0.08 in the study group and 0.81 ± 0.24 in the control group) and frequency of abdominal distention (0.25 ± 0.06 in the study group and 0.72 ± 0.12 in the control group), abdominal circumference (23.18 ± 2.99 in the study group and 24.79 ± 2.99 in the control group) and GRV (0.8 ± 0.10 in the study group and 3.86 ± 1.03 in the control group). These results are similar to those in the study performed by Ardiansyah et al. (2020) and Haghshenas et al. (2020), who revealed that the abdominal massage had a positive effect on the incidence of feeding intolerance in premature infants. In addition, Mojaveri et al. (2020) reported that abdominal massage, which leads to less distension and GRV, is recommended before enteral feeding for very LBW infants and Jin et al. (2020) showed that intervention with abdominal massage might promote weight gain and improve feeding tolerance. Badini Pourazar et al. (2018) detected that touching in premature infants can improve the nutritional tolerance of infants. Ghasemi et al. (2019) demonstrated that abdominal massage effectively diminishes GRV and enhances weight gain in the preterm infants. In addition, Choi et al. (2016) mentioned that massage therapy has potential effects on improving physical growth and gastrointestinal function in premature infants. And, this outcome is consistent with the study performed by Kim and Bang (2018), who reported that enteral feeding improvement massage can assist in achieving earlier full enteral feeding.

However, the results of this study are in contrast to the study by Fazli et al. (2017), who mentioned that abdominal massage was efficient only in the absence of vomiting.

## 6 CONCLUSION

Based on our findings, abdominal massage had a positive impact on the state of alertness after feeding and feeding tolerance indicators, such as the frequency of defecation, vomiting, abdominal distention and abdominal circumference and GRV.

## CONFLICT OF INTEREST

None.

## AUTHOR CONTRIBUTION

All authors participates at all parts of the study.

## Data Availability

All data generated or analysed during this study are included in this published article.
